# Supplementary material for: Modified PID controller for automatic generation control of multi-source interconnected power system using fitness dependent optimizer algorithm
Source: PLoS One. 2020 Nov 20;15(11):e0242428. doi: 10.1371/journal.pone.0242428 (PMC7678963; doi:10.1371/journal.pone.0242428)
Supplement: S1 File — (PDF) [file pone.0242428.s001.pdf]

```
%                                                                       %
%  Authors: Amil et al.%
%                                                                       %%
%                                                                       %
%                                                                       %
%                                                                       %
% this code is an implementation of this paper work                     %
function [dimensions,fitness,upper_bound, lower_bound] =
Select_Functions1(func)


switch func
    case 'AGC'
        fitness = @AGC;
        dimensions = 9;  % number of variable
        lower_bound= -2;
        upper_bound = 2;
% AGC

function Z=AGC(x)

global p1 p2 p3 p4 p5 p6 p7 p8 p9


 p1=x(1);
 p2=x(2);
 p3=x(3);
 p4=x(4);
 p5=x(5);
 p6=x(6);
 p7=x(7);
 p8=x(8);
 p9=x(9);

sim('D:\PHD_Research_2019\Matlab Work\AGC_IPD_two_areaa.slx');
% ZZ=abs(delta_f1)+abs(delta_f2)+abs(delta_ptie); % ITAE
ZZ=(delta_f1.^2)+(delta_f2.^2)+(delta_ptie.^2);

Z=trapz(time,time.*ZZ); % ITSE

end
clear all
clc

global p1 p2 p3 p4 p5 p6 p7 p8 p9

scout_bee_number =60; % Number of search agents

function_name='AGC';
max_iteration = 60;

weightFactor = 0.0;  %equation 2 in the above paper.
```

```
disp(function_name);

tic

 [best_fitness_value, best_scout_bee] = FDO(function_name, max_iteration,
scout_bee_number, weightFactor);


disp('=====================================================')
 disp([' BEST solution   =  '  num2str(best_scout_bee.xs)])
 disp([' BEST fitness    = '   num2str(best_fitness_value)])
 disp(['  Time           =  '  num2str(toc)])


function [ best_fitness_value, best_scout_bee ] = FDO1( function_name,
max_iteration, scout_bee_number, weightFactor )


[dimensions,fitness,upper_bound, lower_bound]  =
Select_Functions1(function_name);


for i=1: scout_bee_number
    scouts(i).xs = lower_bound+rand(1,dimensions)*(upper_bound-lower_bound);
    scouts(i).pace = 0.0;
end


for iterate = 1 : max_iteration

    for s=1 :scout_bee_number
        current_bee = scouts(s);
        best_bee =  getBestScoutBee(scouts); % find global Best bee
        %find current bee fitness_weight fw
        if fitness(best_bee.xs) ~= 0
            fitness_weight = fitness(best_bee.xs)/fitness( current_bee.xs) -
weightFactor;
        end
        for d=1 : dimensions
            x = current_bee.xs(d);
            pace = 0.0;
            random = Levy(1);
            distance_from_best_bee = best_bee.xs(d)- x;
            if fitness_weight == 1
                pace = x * random;
            elseif fitness_weight ==0
                pace = distance_from_best_bee * random;
            else
                pace = (distance_from_best_bee * fitness_weight);
                if random < 0
                    pace = pace * -1;
                end
            end
            x = x +pace;
```

```
            x = getIntoBounderyLimit(x);
            tempBee.xs(d) = x;
            tempBee.pace(d) = pace;
        end
        if fitness(tempBee.xs) < fitness( current_bee.xs)
            current_bee = tempBee;
        elseif  size(current_bee.pace, 2) > 1
            for m=1 : dimensions
                x = current_bee.xs(m);
                distance_from_best_bee = best_bee.xs(m)- x;
                x = current_bee.xs(m) +
(distance_from_best_bee*fitness_weight)+current_bee.pace(m);
                 x = getIntoBounderyLimit(x);
                tempBee.xs(m)= x;
            end
            if fitness(tempBee.xs) < fitness( current_bee.xs)
                current_bee = tempBee;
            end
        else
            for k=1 : dimensions
                x = current_bee.xs(k);
                random = Levy(1);
                x= x + x*random;
                 x = getIntoBounderyLimit(x);
                tempBee.xs(k)= x;
            end
            if fitness(tempBee.xs) < fitness( current_bee.xs)
                current_bee = tempBee;
            end
        end
          scouts(s)= current_bee;
    end
    iterate
    fitness(best_bee.xs)
end

% for rr=1: scout_bee_number
%     fitness(scouts(rr).xs)
% end

best_scout_bee = getBestScoutBee(scouts);
best_fitness_value= fitness(best_scout_bee.xs);



    function max_bee =  getBestScoutBee(scouts)
        max_bee = scouts(1);
        for n=2: scout_bee_number
            if fitness(max_bee.xs) >  fitness(scouts(n).xs)
                max_bee =  scouts(n);
            end
        end

    end
```

```
    function x= getIntoBounderyLimit(x)
        if x > upper_bound
            x = upper_bound * Levy(1);

        elseif x < lower_bound
            x = lower_bound * Levy(1);
        end
    end
end
```
