# Supplementary material for: Modified PID controller for automatic generation control of multi-source interconnected power system using fitness dependent optimizer algorithm
Source: PLoS One. 2020 Nov 20;15(11):e0242428. doi: 10.1371/journal.pone.0242428 (PMC7678963; doi:10.1371/journal.pone.0242428)
Supplement: Attachment — Submitted filename: Final_PLOS_ONE_Chang.pdf [file pone.0242428.s002.pdf]

Modified PID Controller for Automatic Generation Control of Multi-Source Interconnected Power System Using Fitness Dependent Optimizer Algorithm

Amil Daraz[1◑], Suheel Abdullah[1‡], Ihsan Ul Haq[1‡], Khan Bahadar Khan[2‡], Ghulam Fareed [1◑], Farhan Zafar [1◑],

1 Department of Electrical Engineering, Faculty of Engineering and Technology, International Islamic University, Islamabad 44000, Pakistan.

2 Department of Telecommunication Engineering, Faculty of Engineering and Technology, The Islamia University of Bahawalpur, Pakistan.
◑These authors contributed equally to this work.
‡These authors also contributed equally to this work.
¶Membership list can be found in the Acknowledgments section.
* amil.phdee108@iiu.edu.pk

Abstract

In this paper, a modified form of the Proportional Integral Derivative (PID) controller known as the Integral- Proportional Derivative (I-PD) controller is developed for Automatic Generation Control (AGC) of the two-area multi-source Interconnected Power System (IPS). Fitness Dependent Optimizer (FDO) algorithm is employed for the optimization of proposed controller with various performance criteria including Integral of Absolute Error (IAE), Integral of Time multiplied Absolute Error (ITAE), Integral of Time multiplied Square Error (ITSE), and Integral Square Error (ISE). The effectiveness of the proposed approach has been assessed on a two-area network with individual source including gas, hydro and reheat thermal unit and then collectively with all three sources. Further, to validate the efficacy of the proposed FDO based PID and I-PD controllers, comprehensive comparative performance is carried and compared with other controllers including Differential Evolution based PID (DE-PID) controller and Teaching Learning Based Optimization (TLBO) hybridized with Local Unimodal Sampling (LUS-PID) controller. The comparison of outcomes reveal that the proposed FDO based I-PD (FDO-I-PD) controller provides a significant improvement in respect of Overshoot (Osh), Settling time (Ts), and Undershoot (Ush). The robustness of an I-PD controller is also verified by varying parameter of the system and load variation.

1 Introduction

The electrical power system consist of highly complex structures having various network of varied loads are interconnected. The basic purpose of AGC is to provide desired amount of power within satisfactory quality to entire users. The system will be stable when there is an equilibrium between generated power and consumers load. Since, the consumers load normally changes, the active power drawn from the generator increases which reduce the speed of generator or turbine due to variation in frequency. The modern power system comprises of numerous network areas which are connected to transmission lines via tie-lines. AGC plays a key role to sustain the exchange of power

between the control regions via tie-lines and retain the frequency at predetermined value [1, 2].

Automatic Generation Control (AGC) performs a significant contribution in maintaining the stability of the power system. In this regards, substantial consideration has been paid by researches to deal with the Load Frequency Control (LFC). However, initially, most of the work has been performed in the single area network. For instance, the authors in [3, 4] considered a single area network of hydro power generation and reheat thermal power respectively. On the other hand some of the authors worked in single source generation of multi- area IPS and utilized different control techniques. For example Satheeshkumar and Shivakumar in [5] used Ant Lion Optimization (ALO) method for the tuning of Proportional Integral (PI) controller of three area IPS. The authors consider single source of thermal generation units for area-1, hydro units for area-2 and Photovoltaics (PV) for area-3. The outcomes yielded from the proposed method are compared with Genetic Algorithm (GA), Bat Inspired Algorithm (BIA) and PSO. Similarly, the works related to the single source multi-area are presented in reference [6–10].

In past few decades, numerous control techniques have been employed for the AGC of single as well as multi-area with multi-source IPS. Mohanty et al. [11] have deliberate LFC of single area with multi-source including hydro, gas and thermal units by employing I/PI/PID controllers. The DE optimization algorithm is considered in order to tune the proposed controller gains. The authors also extended the research work to two-area diverse power generation systems. The authors in [12] used TLBO with PD-PID controller for two- area network with multi-source system considering hydro, gas and thermal generation units. Patel et al. in [13] suggested a Fractional Order (FO) fuzzy PID controller for two area IPS considering three generation units in one area and three sources in other area. The gain of the controllers are optimized with Ant Lion Optimization (ALO) method.

The literature survey reveals that the nature-inspired metaheuristic optimization algorithms have received tremendous attention from researchers due to their strengths and capabilities to solve numerous complex optimization problems in engineering. These techniques have also been successfully employed for the tuning of controller parameters. In this regard, many authors have employed metaheuristic optimization algorithms based control techniques for AGC. Of these methods, authors have utilized Genetic Algorithm (GA) [14], (PSO) [15], (DE) [16], Improved-Ant Colony Optimization (I-ACO) [17], Firefly Algorithms (FA) [18, 19], Fruit Fly Algorithm (FFA) [20],Improved Grey Wolf Optimization (IGWO) [21], Teaching Learning Base Optimization (TLBO) [22], Symbiotic Organisms Search Algorithm (SOSA) [23], Salp Swarm Algorithm (SSA) [24], Imperialist Competitive Algorithm (ICA) [25], Sine Cosine Algorithm (SCA) [26] and Backtracking Search Algorithm (BSA) [27]. While some of the authors have also applied hybrid techniques like improved firefly with Pattern Search (IF-PS) [28], PSO hybrid with Gravitational Search Algorithm (PSO-GSA) [29], TLBO hybridized with Local Unimodal Sampling (TLBO-LUS) [30], PSO hybridized with Chemical Reaction Optimization (HPSO-CRO) [31], and Hybrid PSO with Levy Flight algorithm (HPSO-LF) [32].

In this paper, FDO algorithm based PID (FDO-PID) and I-PD (FDO-I-PD) controllers are designed for the AGC of multi-source multi-area IPS. In the proposed control model each area contains three different generation sources including hydro, reheat thermal and gas. The efficiency of the proposed controllers are first assessed on a two-area network with individual source i.e gas, hydro and reheat thermal and then collectively with all three sources. To quantify efficacy of the proposed controller's detailed comparative performance is made with the results obtained by DE-PID, TLBO-PID, LUS-PID, LUS-TLBO-PID, FA-PID, FA-I-PD, PSO-PID, PSO-I-PD and

TLBO-I-PD.

The respite of the research work is structured as follows: Section 2 represents the material and methods followed by controller structure and optimization technique, While in section 3 implementation and results are demonstrated. The presented research work is concluded in section 4.

2 Materials and methods

2.1 Controller Structure

In early AGC method, the integral controller had been used to control the system frequency and tie-lines power. However, due to its slower time response, the researchers used Proportional Integral (PI) controller which have the advantages of its low cost, simple structure and faster time response. The poor dynamic performance of PI controller has been improved by PID controller and their modified form I-PD controller which are nowadays very commonly used in practical [33]. In I-PD controller, the proportional parameter and the derivative parameter are put in feedback form while, integrator parameter are put in feed forward direction, as depicted in Fig 2, while in PID controller all the parameters are put in feed forward direction which are depicted in Fig 1.

Fig 1. Structure of PID Controller

Fig 2. Structure of I-PD Controller

The input of PID /I-PD controller for area 1 and 2 is specified by Area Control Error (ACE).

$$ACE_1 = \beta_1 \Delta F_1 + \Delta P_{tie12} \quad (1a)$$

$$ACE_2 = \beta_1 \Delta F_2 + \Delta P_{tie21} \quad (1b)$$

where β_1 and β_2 represents biased parameters of frequency for area-1 and 2 respectively. ΔF_1 and ΔF_2 represents the frequency variation for area-1 and 2 respectively. Similarly, ΔP_{tie12} shows the tie line variation from area-1 to area- 2 and ΔP_{tie12} represent the tie line variation from area-2 to area-1.

To optimize the parameters of a controller, one of the essential steps is to determine the objective function. In this paper four different performance criteria, i.e., ITSE, ISE, ITAE and IAE are applied to verify the system performance and are given in below equations.

$$ITSE = \int_0^T t[\Delta F_1^2 + \Delta F_2^2 + \Delta P_{tie12}^2]dt \quad (2a)$$

$$ISE = \int_0^T [\Delta F_1^2 + \Delta F_2^2 + \Delta P_{tie12}^2]dt \quad (2b)$$

$$ITAE = \int_0^T t[|\Delta F_1| + |\Delta F_2| + |\Delta P_{tie12}|]dt \quad (2c)$$

$$IAE = \int_0^T [|\Delta F_1| + |\Delta F_2| + |\Delta P_{tie12}|]dt \quad (2d)$$

2.2 Fitness Dependent Optimizer

Several optimization methods are introduced by researchers in the field of power system to optimize the gains of the controllers by determination of fitness function. A nature-inspired metaheuristic computational technique known as Fitness Dependent Optimizer (FDO) is deployed in this work to tune the various gains of the proposed controller. FDO starts from the initialization population of scout randomly in search space $K_m; m = 1, 2, 3, \ldots\ldots, n$. The number of the scout bees were equal to population size and each scout contains three parameters known as K_p, K_i and K_d denote the gains of PID/I-PD Controller. Each scout bee denotes a new exposed hive (solution). Scouts bee are randomly searching more position to find best solutions. A previously discovered hive is ignored when new search space is find out. So each time the algorithm finds a better new solution the previous one is discarded. Moreover, if the scout bee does not find the best solutions by moving forward, then it comes back to its previous direction hoping for optimal solutions. However, if the prior solution is unable to provide a better new solution then the existing solution will be considered as the best solution that has been discovered yet. During random movement of the scout bees, each time adding pace to the present position, the scout hopes to determine the best solution. The results are compared with global best and hence it is repeated until the optimal solution not achieved or generation is stopped The movement of scouts is represented as below:

$$k_{m,t+1} = k_{m,t} + p, \tag{3}$$

where m denotes current search agent, t denotes current iteration, K indicate scout bees and pace p is the movement rate and direction. Generally, pace p depends on Fitness Weight w_f and can be articulated as follows:

$$w_f = |\frac{k^*_{m,t,f}}{k_{m,t,f}}| - \alpha \tag{4}$$

where $k^*_{m,t,f}$ represents the value of the best global solution, $k_{m,t,f}$ is the value of current solution and α is a weight factor which is used for the controlling of w_f and its value is either 1 or 0. If the value of α is 1 then, it represent high level of convergence while if the value is 0 then there is no effect on equation (4) but often it provides more stable search. The value of w_f should be in the range of [1, 0]. But in some cases it may be equal to 1, for instance, when the current and global best solution are equal. The value of w_f will be equal to 0 when $k^*_{m,t,f}$ is equal to 0. Finally, the case $k_{m,t,f} = 0$ should be ignored. Hence, the rules given below must be considered:

$$p = Rk_{m,t,f}; \quad \text{if} \quad w_f = 0 \; OR \; w_f = 1 \; OR \; k_{m,t,f} = 0, \tag{5a}$$

$$p = \begin{cases} w_f(k_{m,t,f} - k^*_{m,t,f}) - 1 & \text{if} \quad w_f < 1 \; AND \; w_f > 0 \; AND \; R < 0 \\ w_f(k_{m,t,f} - k^*_{m,t,f}) & \text{if} \quad w_f < 1 \; AND \; w_f > 0 \; AND \; R \geq 0 \end{cases} \tag{5b}$$

where R is the random number in the range of $[1, -1]$. The elementary steps of the FDO are shown in the algorithm 1.

3 Implementation and Results

In this section FDO-I-PD and FDO-PID controllers are considered and employed for a two-area IPS with three different generation units to assess their performance. Further, to evaluate the performance of these controllers, a two-area IPS with a single generation unit including hydro, reheat thermal and gas is considered and then the same two-area network with all three sources is investigated via simulations with 1% step load

Algorithm 1 : Fitness Dependent Optimizer

Cost function $J(.)$;

Generate scout bee population $k_{m,t}; m = 1, 2, \cdots, n$

While Boundary not reached **do**

for all For each scout bee $k_{m,t}$ **do**

$k^*_{m,t} \leftarrow k_{m,t}$

$R \leftarrow r \in [-1 \;\; 1]$ %random walk

if$k_{m,t,f} == 0$ then

$w_f = 0$

else

$w_f \leftarrow |\frac{k^*_{m,t,f}}{k_{m,t,f}}| - \alpha$

end if

if$w_f == 0 OR w_f == 1$ then

$p \leftarrow R \; k_{m,t}$

else

if$R \geq 0$

$p \leftarrow_f (k_{m,t,f} - k^*_{m,t,f}) - 1$

else

$p \leftarrow w_f(k_{m,t,f} - k^*_{m,t,f})$

end if

end if

$K_{m,t+1} \leftarrow K_{m,t} + p$

If$K_{m,t+1,f} < k_{m,t,f}$ then

Save $k_{m,t+1,f}$

Save p

else

$K_{m,t+1} \leftarrow K_{m,t} + p$

if$K_{m,t+1,f} < K_{m,t,f}$ then

Save $k_{m,t+1,f}$

Save p

else keep current position

endIf

endIf

end for

end while

perturbation (SLP). In prvious work, four various performance criteria are used ITSE, ISE, ITAE, and IAE. However, ITSE [15,29], ISE [24,25] and ITAE [11,13] are mostly used for AGC. For the comparison among various performance criteria Eq (2a)- Eq (2d) are executed in Matlab and identified that ITSE provides minimum error as compared to others criteria which are show in Table 2. Hence, ITSE criteria is preferred as an objective function to tune the gains of controller. Further, for the sake of comparison three other methods such as PSO, TLBO and FA are used for tuning of I-PD/ PID controller. The convergence rate for different methods using ITSE criteria is depicted in Fig 3. The time response performance is evaluated by comparing with DE-PID, LUS-PID, LUS-TLBO-PID fuzzy based LUS-TLBO-PID controllers. The parameters of FDO is chosen from Appendix Table 11. For simulation a population of 30 and generation of 60 numbers are considered. The optimization was performed by 30 times for each algorithm and the best optimal gains are picked during optimization which are specified in the Table 1, 4, 6, and 8 for reheat thermal unit, hydro, gas and multi-source unit respectively.

Fig 3. Rate of convergence for different Algorithms

Table 1. Optimum gains of PID /I-PD controllers optimized with different methods for reheat thermal unit.

Controller with Techniques	Reheat Thermal Power		
	K_p	K_i	K_d
FDO-PID	0.260	1.903	-1.680
FDO-I-PD	0.340	1.000	-0.670
PSO-PID	1.023	1.010	-0.223
PSO-I-PD	1.305	0.011	-0.435
FA-PID	0.350	0.001	0.450
FA-I-PD	-0.030	0.600	2.800
TLBO-PID	0.450	1.230	-0.080
TLBO-I-PD	1.700	0.900	-0.203

Table 2. Comparative performance for different indices criteria.

Controller with Techniques	Performance Indices			
	ITSE	*ITAE*	*ISE*	*IAE*
FDO-PID	0.000056	0.000093	0.00067	0.0147
FDO-I-PD	0.000026	0.000019	0.00056	0.0036
PSO-PID	0.000130	0.000210	0.00230	0.0096
PSO-I-PD	0.000190	0.000560	0.00980	0.0663
FA-PID	0.000350	0.000100	0.00450	0.0023
FA-I-PD	0.000040	0.000080	0.00470	0.0196
TLBO-PID	0.000055	0.000023	0.00230	0.0210
TLBO-I-PD	0.000070	0.000090	0.00120	0.0020

3.0.1 Two-Area Reheat Thermal Power System

The diagram of reheat thermal power with two-area units is presented in Fig 4. The area 1 contains Transfer Functions (TF) of the governor $\frac{1}{T_t s+1}$, reheat $\frac{K_1 T_r s+1}{T_r s+1}$ and turbine $\frac{1}{T_1 s+1}$ of the reheat thermal power system, where K_t represents thermal constant, R_{th1} denotes the droop constant of area 1, ΔP_D indicates change in load perturbation and ΔF_1 is change of frequency in area 1. Similarly, area 2 comprises of TF of the governor,

reheat and turbine of the reheat thermal power system, where R_{th2} symbolizes the droop constant of area 2 and ΔF_2 represents the frequency change in area 2.

Fig 4. Two-area model with reheat thermal power system.

The TF diagram for two-area reheat thermal IPS are developed in Matlab/Simulink using the values of the gains from Appendix Table 10. The system is evaluated with I-PD/ PID controllers with 1% SLP at $t = 0s$. Fig (5-10) depicts the responses of load frequency in each area and tie-line power. In pursuance to examine the effectiveness of the FDO-PID and FDO-I-PD control strategies, the output responses are compared with several other algorithms based PID and I-PD controllers including PSO-PID, PSO-I-PD, FA-PID, FA-I-PD,TLBO-PID, and TLBO-I-PD. The comparison of transient response specifications are quantified and presented in Table 3.

Fig 5. Results for reheat thermal unit in area 1 with PID Controller.

Fig 6. Results for reheat thermal unit in area 2 with PID Controller.

Fig 7. Results for reheat thermal unit in area 1 with I-PD Controller

Fig 8. Results for reheat thermal unit in area 2 with I-PD Controller

Fig 9. Results for reheat thermal unit of tie-line power with PID Controller

Fig 10. Results for reheat thermal unit of tie-line power with I-PD Controller

The comparison of results from Fig 5 and Table 3 reveal that FDO-PID controller completely eliminates overshoot O_{sh} as compared to PSO, FA and TLBO based PID controller which is dire need of a controller for the system stability. Further, settling time (T_s) and undershoot (U_{sh}) yielded by FDO-PID are better than PSO/FA/TLBO based PID controller. The results shown in Fig 6 further reveal that FDO-PID controller provides less O_{sh} than PSO-PID however, at the cost of slight increase in T_s, but better than FA-PID and TLBO-PID.

The results given in Fig 7 and 8 further shows that FDO-I-PD controller for both area 1 and area 2 outperform in terms of T_s, O_{sh} and U_{sh} which is less than PSO/FA/TLBO base tuned PID controllers. The results shown in Fig 9 reveals that FDO-PID has lesser O_{sh} as compared to other controllers at the cost of increase in settling time for PSO-PID and FA-PID but still better than TLBO-PID. The outcome given in Fig 10 express the overall superiority of FDO-I-PD than other controllers in all aspects i.e less T_s, O_{sh} and U_{sh}. The overall comparison of the results for area 1 and 2 for reheat thermal power system is quantified and presented in Table 3.

3.1 Two Area Hydro Power System

The transfer function(TF) diagram for hydro power unit with two area is shown in Fig 11 which consist of the Transfer Function (TF) of hydro governor $\frac{1}{T_{gh}s+1}$, penstock

Table 3. Comparative performance between different algorithms for reheat thermal with two-area power system.

Controller with Techniques	Settling Time $T_s s$			Overshoot O_{sh}			Undershoot U_{sh}		
	ΔF_1	ΔF_2	ΔP_{tie}	ΔF_1	ΔF_2	ΔP_{tie}	ΔF_1	ΔF_2	ΔP_{tie}
**FDO-PID**	**2.9**	**9.2**	**10.3**	**0.00000**	**0.00250**	**0.00021**	**-0.00400**	**-0.00200**	**-0.00200**
**FDO-I-PD**	**1.9**	**10.1**	**4.9**	**0.00000**	**0.00012**	**0.00000**	**-0.00050**	**-0.00090**	**-0.00620**
PSO-PID	6.5	9.1	8.4	0.00180	0.01630	0.00160	-0.01000	-0.00690	-0.01010
PSO-I-PD	8.2	14.2	6.2	0.00150	0.00030	0.00930	-0.00680	-0.00130	-0.00910
FA-PID	6.3	10.4	8.7	0.01200	0.00280	0.00081	-0.08400	-0.01700	-0.00500
FA-I-PD	10.9	16.5	9.3	0.00072	0.00025	0.00710	-0.00480	-0.00110	-0.00180
TLBO-PID	6.4	10.2	11.6	0.01300	0.00270	0.00091	-0.06300	-0.01800	-0.00160
TLBO-I-PD	3.8	14.3	8.3	0.00020	0.00310	0.00000	-0.00460	-0.00070	-0.01100

turbine $\frac{T_r s+1}{T_{rh} s+1}$ and transient droop compensation $\frac{-T_w s+1}{0.5T_w s+1}$. In this system, K_h, R_{h1} and R_{h2} are hydral constant, the hydral droop constant of area 1 and 2 respectively.

Fig 11. Two-area model with hydro power unit.

Table 4. Optimum gains of PID /I-PD controllers optimized with different methods for hydro power unit.

Controller with Techniques	Hydro Power system		
	K_p	K_i	K_d
FDO-PID	1.163	1.962	-0.003
FDO-I-PD	1.200	1.012	1.001
PSO-PID	0.962	0.003	0.002
PSO-I-PD	1.012	1.001	1.012
FA-PID	1.002	1.002	0.001
FA-I-PD	1.013	1.012	0.080
TLBO-PID	0.450	0.093	-0.080
TLBO-I-PD	0.700	0.010	-0.230

The TF model of two-area hydro power system have been assessed with FDO-PID and FDO-I-PD. The results are compared with PID and I-PD with other tuning techniques i.e.PSO, TLBO and FA. The results obtained from two-area hydro power system with PID and I-PD controllers are given in Fig (12-17).

Fig 12. Results for hydro power unit in area 1 with PID Controller.

Fig 13. Results for hydro power unit in area 2 with PID Controller.

Fig 14. Results for hydro power unit in area 1 with I-PD Controller

Fig 15. Results for hydro power unit in area 2 with I-PD Controller

Fig 16. Results for hydro power unit of tie-line power with PID Controller

Fig 17. Results for hydro power unit of tie-line power with I-PD Controller

The results obtained from interconnected hydro power system with PID controller are given in Fig 12 which shows the better performance of FDO-PID controller as compared to TLBO/FA/PSO based PID controller. The results in Fig 13 reflect that FDO-PID provides output response with zero overshoot ($O_{sh} = 0.0000$) as compared to PSO-PID ($O_{sh} = 0.0029$), FA-PID ($O_{sh} = 0.0019$) and TLBO-PID ($O_{sh} = 0.0006$) controllers. Similarly, the results also express that FDO-PID yields lesser U_{sh} and T_s as compared to PID controller with other tuning techniques. The results depicted in Fig 14 indicate that FDO-I-PD controller provides excellent results with zero O_{sh} in load frequency of area 1, less settling time and undershoot as associated with other techniques like PSO, FA and TLBO with same controllers. The results illustrated in Fig 15 express that FDO base tuned PID controller have less settling time, undershoot and overshoot than PSO, TLBO and FA base tuned PID controller. However, a minor change of 0.001 in overshoot can be clearly seen than PID based controller optimized with FDO algorithm. From Fig 16 it can be seen that FDO base tuned algorithm have less settling time nonetheless of minor increased in overshoot which is completely eliminated by FDO based tuned I-PD controller which is indicated in Fig 17 but still better than other used techniques in terms of T_s, O_{sh} and U_{sh}. A comprehensive comparison results for two-area hydro power system and change in tie-lines in terms of T_s, O_{sh} and U_{sh} are given in Table 5. The representation of bold values indicates the best results.

Table 5. Comparative performance between different algorithms for two-area hydro power system.

Controller with Techniques	Settling Time T_s			Overshoot O_{sh}			Undershoot U_{sh}		
	ΔF_1	ΔF_2	ΔP_{tie}	ΔF_1	ΔF_2	ΔP_{tie}	ΔF_1	ΔF_2	ΔP_{tie}
**FDO-PID**	**2.83**	**2.30**	**3.20**	**0.00000**	**0.00000**	**0.00020**	**-0.00760**	**-0.00430**	**-0.00023**
**FDO-I-PD**	**2.90**	**5.30**	**4.00**	**0.00000**	**0.00100**	**0.00000**	**-0.00410**	**-0.00190**	**-0.00100**
PSO-PID	7.30	6.40	10.10	0.00290	0.00190	0.00600	-0.06400	-0.00520	-0.01300
PSO-I-PD	6.70	9.80	8.70	0.00600	0.00500	0.00730	-0.00420	-0.00130	-0.00460
FA-PID	4.30	5.60	10.20	0.00190	0.00060	0.00420	-0.04300	-0.00700	-0.00600
FA-I-PD	5.40	10.1	6.80	0.00800	0.00700	0.00490	-0.00930	-0.00150	-0.00430
TLBO-PID	2.40	4.20	4.60	0.00060	0.00056	0.00100	-0.06200	-0.00560	-0.00500
TLBO-I-PD	3.10	6.20	4.40	0.00210	0.00300	0.00000	-0.00420	-0.00170	-0.00900

3.2 Two-Area Gas Power System

The Transfer Function (TF) diagram of two area gas power generation is show in Fig 18. The two-area are interconnected by tie lines whereas, each area indicate the TF of valve position, speed governor, compressor discharge and fuel with combustion reaction. K_g, R_{g1} and R_{g2} denote the gas constant, and gas droop constant for area 1 and 2 respectively.

Fig 18. Two-area model with gas power system.

The TF model of two-area gas power system are established in Matlab/Simulink using Appendix Table 10. The system is assessed with FDO base optimized PID/I-PD controllers to evaluate the achievement of the proposed techniques and their outcomes are compared with some recent optimization algorithms like TLBO, FA and PSO. The results obtained from two-area gas power system for area 1 and 2 with PID and I-PD controllers are given in Fig (19-24).

Table 6. Optimum gains of PID /I-PD controllers optimized with different methods for gas generation unit.

Controller with Techniques	Gas Power system		
	K_p	K_i	K_d
FDO-PID	0.723	0.112	-1.012
FDO-I-PD	0.305	0.811	0.029
PSO-PID	0.962	0.003	0.002
PSO-I-PD	1.060	0.723	0.687
FA-PID	1.002	1.002	0.001
FA-I-PD	0.062	1.100	0.458
TLBO-PID	1.072	1.007	-0.238
TLBO-I-PD	0.723	0.112	-1.012

Fig 19. Results for gas unit in area 1 with PID Controller.

Fig 20. Results for gas unit in area 2 with PID Controller.

Fig 21. Results for gas unit in area 1 with I-PD Controller

Fig 22. Results for gas unit in area 2 with I-PD Controller

Fig 23. Results for gas unit of tie-line power with PID Controller

Fig 24. Results for gas unit of tie-line power with I-PD Controller

In Fig 19 the results reveal that FDO base PID controller completely eliminate the overshoot ($o_{sh} = 0.000$), less settling time ($T_s = 2.1$) and undershoot ($U_{sh} = -0.0048$) as compared to PSO ($O_{sh} = 0.0036$, $T_s = 5.9s$, $U_{sh} = -0.0072$), FA ($O_{sh} = 0.021$, $T_s = 3.9s$, $U_{sh} = -0.0083$), and TLBO ($O_{sh} = 0.019$, $T_s = 5.2s$, $U_{sh} = -0.0049$) base PID controller. The results depicted in Fig 20 express that FDO base tuned PID controller have similar settling time and overshoot but less undershoot than TLBO base tuned PID controller, nonetheless better than remaining techniques i.e. FA and PSO.

The results given in Fig 21 and 22 indicates that FDO base tuned I-PD controller for change in frequency with area 1 and area 2 have good performance in terms of T_s, O_{sh} and U_{sh} which is less than PSO/FA/TLBO base tuned PID controllers. In inclusive comparison of the results for area 1 and 2 of gas power system in settling time, overshoot, undershoot and different performance indices are given in table 9. The outcome shown in Fig 23 articulates that FDO base tuned PID controller for change in tie- line power have better results in all aspects i.e T_S, O_{sh} and U_{sh} from other applied techniques with same controller. From Fig 24 it can observed that FDO base optimized I-PD controller have better result than same controller tuned with PSO/FA/TLBO in terms of lesser O_{sh}, T_S and U_{Sh}. The best results are indicated with bold formats.

3.3 Two-Area Multi-Source Interconnected Power System

The Transfer Function (TF) diagram for multi-source with two-area IPS are depicted in Fig 25. Various power generation sources like reheat thermal, gas power and hydro

Table 7. Comparative performance between different algorithms for two-area gas power system.

Controller with Techniques	Settling Time T_s			Overshoot O_{sh}			Undershoot U_{sh}		
	ΔF_1	ΔF_2	ΔP_{tie}	ΔF_1	ΔF_2	ΔP_{tie}	ΔF_1	ΔF_2	ΔP_{tie}
**FDO-PID**	**2.1**	**10.2**	**3.1**	**0.00000**	**0.00013**	**0.00003**	**0.00730**	**-0.00720**	**-0.00059**
**FDO-I-PD**	**4.0**	**3.9**	**5.2**	**0.00000**	**0.00000**	**0.00000**	**-0.00100**	**-0.00084**	**-0.00047**
PSO-PID	5.9	12.4	10.4	0.03600	0.00390	0.00040	-0.00480	-0.00160	-0.00110
PSO-I-PD	8.7	9.6	5.9	0.00730	0.00201	0.00930	-0.00460	-0.00019	-0.00054
FA-PID	3.9	12.3	7.9	0.00210	0.00320	0.00016	-0.00490	-0.00170	-0.00064
FA-I-PD	6.8	9.2	6.3	0.00490	0.00000	0.00021	-0.00430	-0.00082	-0.00052
TLBO-PID	5.2	10.2	6.8	0.01000	0.00013	0.00017	-0.00830	-0.00080	-0.00061
TLBO-I-PD	4.4	5.9	10.1	0.00000	0.00000	0.00000	-0.00900	-0.00089	-0.00096

power system for each area are collectively applied.

Fig 25. Two-area with multi-source power system.

Table 8. Optimum values of I-PD/ PID controllers tuned with various techniques for power system.

Controller with Techniques	Reheat Thermal Power			Hydro Power System			Gas Power System		
	K_p	K_i	K_d	K_p	K_i	K_d	K_p	K_i	K_d
**FDO-PID**	0.36	**0.23**	**-0.67**	**0.47**	**0.23**	**-0.32**	**1.36**	**0.23**	**0.67**
**FDO-I-PD**	**0.56**	**0.09**	**-0.56**	**1.03**	**0.90**	**0.01**	**1.56**	**0.90**	**0.56**
PSO-PID	0.23	1.00	-0.03	1.96	1.10	0.01	0.23	1.00	0.23
PSO-I-PD	1.19	1.56	0.23	1.63	0.90	0.01	1.19	0.56	-0.23
FA-PID	0.35	0.01	-0.45	1.10	2.00	1.00	0.35	0.01	0.45
FA-I-PD	0.60	1.80	0.45	1.16	1.96	-0.03	0 .60	-1.80	0.45
TLBO-PID	0.45	0.23	0.89	1.07	1.90	0.10	0.45	0.23	-0.23
TLBO-I-PD	0.70	1.90	0.49	2.00	0.90	0.10	0.70	1.90	-0.23

Two-area multi-source interconnected power system have been developed in Matlab Simulink and the values have been used from Appendix Table 10. The system is assessed with FDO base tuned I-PD/ PID controllers and the superiority of the proposed techniques are compared with other techniques i.e TLBO, FA and PSO. The results are depicted in Fig (26-31).

Fig 26. Results for Multi-Source in area 1 with PID Controller.

Fig 27. Results for Multi-Source in area 2 with PID Controller.

Fig 28. Results for Multi-Source in area 1 with I-PD Controller

Fig 29. Results for Multi-Source in area 2 with I-PD Controller

Fig 30. Results for Multi-Source of tie-line power with PID Controller

Fig 31. Results for Multi-Source of tie-line power with I-PD Controller

The results shown in Fig 26 for multi-source interconnected power system of area 1 reveals that PID controller with FDO base algorithm have no overshoot, less settling time and undershoot as associated with other techniques like PSO, TLBO and FA. The results depicted in Fig 27 express that FDO base tuned PID controller have less overshoot and settling time than TLBO and FA base tuned PID controller however at the cost of 0.001 increase in undershoot, but shows better improvement than PSO base tuned PID controller.

The results shown in Fig 28 express that FDO-PID has less settling time T_s, undershoot U_sh and overshoot O_sh than PSO-PID, TLBO-PID and FA-PID. However, a minor change of 0.001 in O_sh can be clearly seen than FDO-PID. The results given in Fig 29 indicates that FDO-I-PD technique for change in frequency of area 2 has no overshoot, less settling time and undershoot as compared with other techniques like PSO-I-PD, FA-I-PD and TLBO-I-PD. The results given in Fig 30 and 31 reflect that FDO-I-PD for change in tie line power have good performance in terms of T_s, O_{sh} and U_{sh} which is less than PSO/FA/TLBO base optimized PID/I-PD controllers. A comprehensive comparison results for two-area multi- source power system and tie lines in terms of T_s, O_{sh} and U_{sh} are given in Table 9. The representation of bold values indicates the best results. the percentage improving comparing with different techniques is shown in bar chart of Fig 32.

Table 9. Comparative Performance between Different Algorithms for two-area Multi-source Power System.

**Controller with Techniques**	**Settling Time** T_s			**Overshoot** O_{sh}			**Undershoot** U_{sh}		
	ΔF_1	ΔF_2	ΔP_{tie}	ΔF_1	ΔF_2	ΔP_{tie}	ΔF_1	ΔF_2	ΔP_{tie}
**FDO-PID**	5.20	12.70	4.30	0.00000	0.00021	0.00020	-0.00047	-0.00130	-0.00056
**FDO-I-PD**	**2.30**	1.65	2.10	0.00000	0.00000	0.00000	-0.00450	-0.00500	-0.00058
PSO-PID	5.90	13.40	9.30	0.00930	0.00250	0.00630	-0.00054	-0.00016	-0.00580
PSO-I-PD	2.90	9.20	6.80	0.00400	0.00400	0.00080	-0.00720	-0.05500	-0.00910
FA-PID	6.30	13.10	7.10	0.00021	0.00036	0.00610	-0.00052	-0.00110	-0.00510
FA-I-PD	4.30	4.10	5.90	0.00000	0.00000	0.00043	-0.00490	-0.00900	-0.00640
TLBO-PID [30]	9.37	3.76	4.76	0.00172	0.00043	0.00017	-0.01972	-0.01279	-0.00307
TLBO-I-PD	**2.20**	2.20	2.20	0.00000	0.00000	0.00011	-0.00530	-0.00500	-0.00130
LUS-TLBO Fuzzy-PID [30]	5.26	2.96	2.36	0.00055	0.00021	0.00008	-0.00895	-0.00301	-0.00096
DE Fuzzy-PID [30]	5.30	2.90	2.60	0.00073	0.00024	0.00009	-0.01297	-0.00589	-0.00160
LUS-TLBO-PID [30]	9.22	3.34	4.98	0.00167	0.00042	0.00015	-0.01689	-0.01133	-0.00289
LUS-PID [30]	9.29	4.62	6.36	0.00185	0.00046	0.00015	-0.02105	-0.01517	-0.00358
DE-PID [11]	13.84	8.35	9.35	0.00203	0.00077	0.00019	-0.02657	-0.02214	-0.00475

Fig 32. Comparison in Sense of Improvement% with Reference DE-PID [11].

3.4 Sensitivity Analysis

The robustness of an I-PD controller is verified by varying the system parameters of two areas multi-generation system within a range of ± 25%. Fitness dependent algorithm (FDO) is employed to check the performance of the proposed controller by varying some parameters of the system from their nominal values such as turbine constant (T_t), droop constant R and governor constant (T_g). The results yielded from sensitivity analysis for

ΔF_1, ΔF_2, and ΔP_{tie} with variation in T_t, R and T_g is depicted in Figure 33-41. It can be observed from results that our proposed controller provides robustness by changing system parameters T_t, R and T_g for ΔF_1, ΔF_2, and ΔP_{tie} respectively within a range of ± 25%. Further, it can be also observed that optimal values of controller gains need not be re-tuned for a wide change of system parameters and load conditions.

Fig 33. Results for variation in R with ΔF_1.

Fig 34. Results for variation in R with ΔF_2.

Fig 35. Results for variation in R with ΔP_{tie}.

Fig 36. Results for variation in T_g with ΔF_1.

Fig 37. Results for variation in T_g with ΔF_2.

Fig 38. Results for variation in T_g with ΔP_{tie}.

Fig 39. Results for variation in T_t with ΔF_1.

Fig 40. Results for variation in T_t with ΔF_2.

Fig 41. Results for variation in T_t with ΔP_{tie}.

4 Conclusion

In this paper, PID and I-PD controllers have been designed and successfully implemented to handle Automatic Generation Control (AGC) of multi-source with multi-area Interconnected Power System (IPS). Fitness Dependent Optimizer (FDO) algorithm has been used to attain the gains of these controllers. The effectiveness of FDO based controllers i.e. FDO-PID and FDO-I-PD are evaluated on two-area with reheat thermal, gas and hydro power system individually and then collectively with all three generation units present in the system. The transient response performance achieved by the designed controllers with 1% step load perturbation are presented and quantified in detail via simulations and compared with several other controller techniques. The comparison of results evidently shows that FDO-PID and FDO-I-PD controllers provide superior results in respect of Settling time (Ts), overshoot (Osh), and undershoot (Ush) for thermal reheat, gas, hydro and multi-source power generation. It is observed that FDO-I-PD as compared to DE-PID completely eliminates Osh in load frequency of both areas and in tie-line power, while 83.38%, 80.24%, and 77.54% improvement in Ts in area-1 and area-2 load frequency, and tie-line power are achieved respectively. Similarly, FDO-I-PD also provides an improvement of 83.1%, 77.4%, and 87.8% in Ush as compared DE-PID. The results further show that FDO- I-PD as

compared to LUS-PID provides significant improvement of 75.24%, 64.28% and 66.98% in Ts of load frequency forΔF_1, ΔF_2, and ΔP_{tie} respectively, while an improvement in Ush of 63.23%, 49.43% and 57.58% are achieved for load frequency of ΔF_1, ΔF_2, and ΔP_{tie} respectively. The supremacy of FDO based PID and I-PD controllers proposed in this work clearly demonstrate the capability of these controllers to tackle the automatic generation control problem effectively with oscillation-free and quick response.

Author Contributions

Conceptualization: Suheel Abdullah Malik, Ihsan Ul haq, Khan Bahadar Khan. **Formal analysis**: Amil Daraz, Suheel Abdullah Malik. **Methodology**: Amil Daraz, Suheel Abdullah Malik. **Software**: Amil Daraz. **Supervision**: Suheel Abdullah Malik, Ihsan Ul haq. **original draft**: Amil Daraz, Suheel Abdullah Malik. **Writing – review editing**: Suheel Abdullah Malik, Farhan Zafar, Ghulam Fareed Laghari, Khan Bahadar Khan.

Appendix

Table 10. Parameter Setting for Two-Area Interconnected Power System [13].

Parameters	Values	Parameters	Values	Parameters	Values
β_1, β_2	0.431 MW/Hz	$R_{th1}, R_{th2}, R_{hy1}, R_{hy2}, R_{g1}, R_{g2}$	2.40 Hz/p.u	T_{gh}	0.080 s
T_t	0.30 s	K_1	0.30	T_r	10 s
K_P	68.95	T_p	11.490 s	T_{12}	0.0430
a_{12}	-1	T_w, y_c	1 s	T_{rs}	5 s
T_{rh}	28.70 s	T_{gh}, T_{cd}, T_{DC}	0.20 s	x_c	0.60 s
K_g	0.1304	K_{DC}, x_g	1	b_g	0.050 s
K_t	0.5434	T_{cr}	0.010 s	T_F	0.230 s
K_h	0.3268	-	-	-	-

Table 11. Values of Fitness Dependent Optimizer parameters.

Parameters	Values	Parameters	Values	Parameters	Values
Number of Population N_P	30	Number of generation N_g	60	Lower bound L_b	-2
Upper bound U_b	2	Number of dimensions N_d	9	Weight Factor γ	0.0
random number α	[-1, 1]		-	-	-

References

1. Arya Y. A novel CFFOPI-FOPID controller for AGC performance enhancement of single and multi-area electric power systems. ISA Trans. 2020;100:126-135. doi:10.1016/j.isatra.2019.11.025.

2. Alhelou HH, Hamedani-Golshan ME, Zamani R, Heydarian-Forushani E, Siano P. Challenges and Opportunities of Load Frequency Control in Conventional, Modern and Future Smart Power Systems: A Comprehensive Review. Energies 2018, 11.

3. Sutterfieldand J, Caprez G, Hanelineand J, Bergman A. Automatic generation control for hydro System.IEEE Trans. Energy Convers 1988.
4. Nanda J, Mangla A, Suri S. Some new findings on automatic generation control of an interconnected hydrothermal system with conventional controllers. IEEE Transactions on Energy Conversion 2006, 21, 187–194.
5. Satheeshkumar R, Shivakumar R. Ant Lion Optimization Approach for Load Frequency Control of Multi-Area Interconnected Power Systems. Circuits and Systems, 7, 2357-2383.2006.
6. Kusic GL, Sutterfield JA, Caprez AR, Haneline JL, and Bergman BR. Automatic generation control for hydro systems. IEEE Trans. Energy Convers. vol. 3, no. 1, pp. 33–39, Mar. 1988.
7. Chen C, Zhang K, Yuan K, Teng X. Tie-Line Bias Control Applicability to Load Frequency Control for Multi-Area Interconnected Power Systems of Complex Topology. Energies 2017, 10.
8. Gozde H, Taplamacioglu MC. Automatic generation control application with craziness based particle swarm optimization in a thermal power system. Int J Electr Power Energy Syst 2011; 33: 8–16.1.
9. Rani P, Jaswal R. Automatic load frequency control of multi - area power system using ANN controller and Genetic algorithm. International Journal of Engineering Trends and Technology (IJETT) 2016, 4.
10. Singh SP, Prakash T, Singh VP. Analytic hierarchy process based automatic generation control of multi-area interconnected power system using Jaya algorithm. Eng Appl Artif Intell 2017; 60: 35–44..
11. Mohanty B, Panda S, and Hota P. Controller parameters tuning of differential evolution algorithm and its application to load frequency control of multi-source power system. International Journal of Electrical Power Energy Systems 2014, 54, 77 – 85.
12. Debnat MK, Patel NC, Mallick RK. Optimal base PD-PID controller for automatic generation control of multi-source tuned by teaching learning base optimization algorithm. IEEE conference 2016, pp. 77—-85.
13. Patel NC, Sahu BK, Bagarty DP, Das P, Debnath MK. A novel application of ALO-based fractional order fuzzy PID controller for AGC of power system with diverse sources of generation International Journal of Electrical Engineering Education · February 2019. DOI: 10.1177/0020720919829710.
14. Daneshfar F, Bevrani H. Multiobjective design of load frequency control using genetic algorithms. Int J Elect Power Energy System 2012, 54, 257-263.
15. Morsali J, Zare K, Hagh MT. Performance comparison of TCSC with TCPS and SSSC controllers in AGC of realistic interconnected multi-source power system. Ain Shams Engineering Journal 2016, 7, 143 – 158.
16. Panigrahi TK, Behera A, Sahoo AK. Novel approach to Automatic Generation Control with various Non-linearities using 2-degree-of-freedom PID controller. Energy Procedia 2017, 138, 464 – 469.

17. Chen G, Li Z, Zhang Z. Li. S, An Improved ACO Algorithm Optimized Fuzzy PID Controller for Load Frequency Control in Multi Area Interconnected Power Systems, IEEE Access, vol. 8, pp. 6429-6447, 2020, doi: 10.1109/ACCESS.2019.2960380..

18. Pradhan PC, Sahu RK, Panda S. Firefly algorithm optimized fuzzy PID controller for AGC of multi-area multi-source power systems with UPFC and SMES. Engineering Science and Technology, an International Journal 2016, 19, 338 – 354.

19. Sufyan M, Abd Rahim N, Tan C, Muhammad MA, Sheikh Raihan SR (2019) Optimal sizing and energy scheduling of isolated microgrid considering the battery lifetime degradation. PLoS ONE 14(2): e0211642. https://doi.org/10.1371/journal.pone.0211642.

20. Shankar R, Kumar A , Raj U, Chatterjee K. Fruit fly algorithm-based automatic generation control of multi-area interconnected power system with FACTS and AC/DC links in deregulated power environment," Int Trans Electrical Energy Syst., vol. 29, pp. 1–25,2019.

21. Sahu1 PC, Prusty RC, Panda S, "Improved-GWO designed FO based type-II fuzzy controller for frequency awareness of an AC micro grid under plug in electric vehicle," Journal of Ambient Int and Humanized Computing, March 2020,doi: 10.1007/s12652-020-02260-z

22. Sahu RK, Panda S, Rout UK, Sahoo DK. Teaching learning based optimization algorithm for automatic generation control of power system using 2-DOF PID controller. International Journal of Electrical Power and Energy Systems 2016, 77, 287 – 301.

23. Guha D, Roy PK, Banerjee S. Symbiotic organism search algorithm applied to load frequency control of multi-area power system. Energy Systems 2018, 9, 439–468.

24. Hasanien HM. Fergany EL, Salp swarm algorithm-based optimal load frequency control of hybrid renewable power systems with communication delay and excitation cross-coupling effect, Electr. Power Syst. Res., vol. 176, Nov. 2019, Art. No. 105938.

25. Arya y. Improvement in automatic generation control of two-area electric power systems via a new fuzzy aided optimal PIDN-FOI controller. ISA Transactions 2018, 80, 475 – 490.

26. Tasnin W, Saikia LC, Maiden application of an sine-cosine algorithm optimized FO cascade controller in automatic generation control of multi-area thermal system incorporating dish-stirling solar and geothermal power plants, IET Renewable Power Generation 2018; 12(5):585–97..

27. Guha D, Roy PK, Banerjee S. Application of backtracking search algorithm in load frequency control of multi-area interconnected power system. Ain Shams Engineering Journal 2018, 9, 257 – 276.

28. Rajesh KS, Dash SS, Rajagopal R. Hybrid improved firefly -pattern search optimized fuzzy aided PID controller for automatic generation control of power systems with multi-type generations, Swarm Evol Comput., vol. 44, pp. 200-211, Feb. 2019.

29. Veerasamy v et al., A Hankel Matrix Based Reduced Order Model for Stability Analysis of Hybrid Power System Using PSO-GSA Optimized Cascade PI-PD Controller for Automatic Load Frequency Control. in IEEE Access, vol. 8, pp. 71422-71446, 2020, doi: 10.1109/ACCESS.2020.2987387.

30. Sahu Bk, Pati Tk, Nayak JR, Panda S, and Kumar S. A novel hybrid LUS–TLBO optimized fuzzy PID controller for load frequency control of multi-source power system. Electrical power and Energy System 2016, pp. 58–69.

31. Mohanty B, Hota P. A hybrid chemical reaction-particle swarm optimisation technique for automatic generation control. Journal of Electrical Systems and Information Technology 2018, 5, 229 – 244.

32. Barisal AK, Panigrahi TK, Mishra S. A Hybrid PSO-LEVY Flight Algorithm Based Fuzzy PID Controller for Automatic Generation Control of Multi Area Power Systems: Fuzzy Based Hybrid PSO for Automatic Generation Control. Int. J. Energy Optim. Eng. 2017, 6, 42–63.

33. Odili JB, Mohmad Kahar MN, Noraziah A (2017) Parameters-tuning of PID controller for automatic voltage regulators using the African buffalo optimization. PLoS ONE 12(4): e0175901. https://doi.org/10.1371/journal.pone.0175901.
